# Supplementary material for: BBS Proteins Affect Ciliogenesis and Are Essential for Hedgehog Signaling, but Not for Formation of iPSC-Derived RPE-65 Expressing RPE-Like Cells
Source: Int J Mol Sci. 2021 Jan 29;22(3):1345. doi: 10.3390/ijms22031345 (PMC7866284; doi:10.3390/ijms22031345)
Supplement: Supplementary file 1 [file ijms-22-01345-s001.pdf]

Supplementary figures

BBS proteins affect ciliogenesis and are essential for hedgehog signaling, but not for formation of iPSC-derived RPE-65 expressing RPE-like cells

Caroline Amalie Brunbjerg Hey, Lasse Jonsgaard Larsen, Zeynep Tümer, Karen Brøndum-Nielsen, Karen Grønskov, Tina Duelund Hjortshøj and Lisbeth Birk Møller

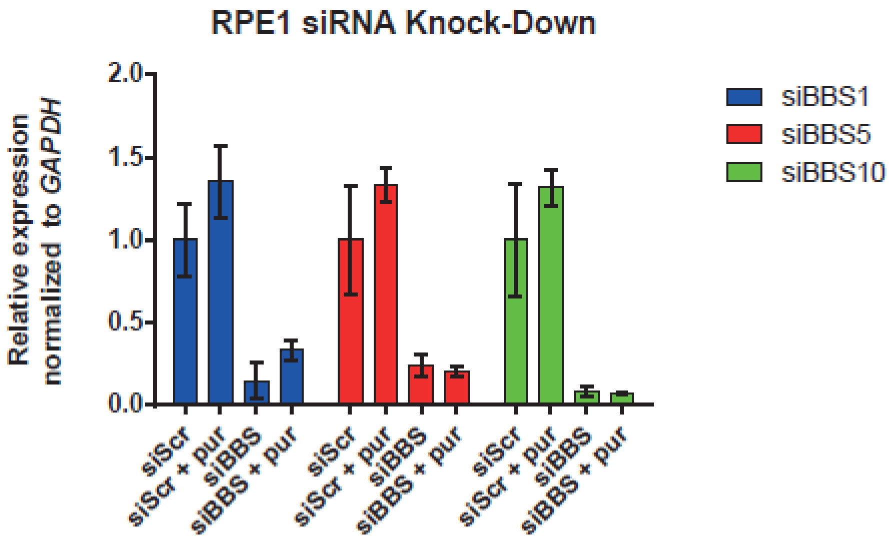

Supplementary Figure 1:

Knock-down efficiency of the used siRNA targeting BBS1, BBS5 and BBS10 in RPE1 cells was assessed through qPCR. n=3 biological replicates. Colors represent target of taqman probe (BBS1, BBS5 and BBS10), error bars the standard deviation. Data was calculated using the delta CT method with normalization to *GAPDH* and siSCR.

Ctrl

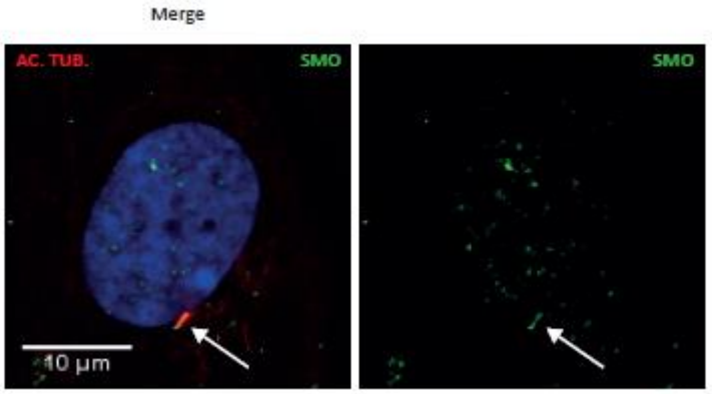

Supplementary Figure 2:

IFM analysis of control fibroblasts (here Ctrl.A), grown in serum reduced media for 48 hours in the presence of pur for the last 24 hours. The cells are labeled with anti AC-TUB (primary cilia marker) and anti SMO antibody. Nuclei were visualized with DAPI staining. Scale bars 10µM.
